# Buying-shopping disorder, impulsivity, emotional dependence and attachment in adolescents

**DOI:** 10.1007/s12144-023-04425-3

**Published:** 2023-02-24

**Authors:** Nerea Etxaburu, Janire Momeñe, Marta Herrero, María Dolores Chávez-Vera, Leticia Olave, Itziar Iruarrizaga, Ana Estévez

**Affiliations:** 1 University of Deusto, Bilbao, Spain; 2 Technical University of Manabí, Portoviejo, Ecuador; 3 Complutense University of Madrid, Madrid, Spain; 4 Psychology Department, University of Deusto, Apartado 1, 48080 Bilbao, Spain

**Keywords:** Buying-shopping disorder, Impulsivity, Emotional dependence, Attachment

## Abstract

In recent years, several studies have shown that the incidence of buying-shopping disorder (BSD) is increasing. Impulsivity is one of the factors involved in its aetiology and is related to emotional dependence. In addition, early affective deprivation may trigger emotional dependence. The aims of the present study weresal: to compare the types of attachment, levels of emotional dependence, impulsivity and BSD according to sex; to determine whether the proposed relational model is fulfilled; and analyse possible differences in this model in terms of the sample’s sex. The sample consisted of 1498 adolescents (53.8% men and 46.2% women) from Ecuador whose age ranged from 14 to 18 years (*M* = 15.77, *SD* = 1.21). The results showed that a preoccupied attachment style is indirectly related to a higher risk of BSD due to emotional dependence when impulsivity levels are medium or high because emotional dependence is moderated by impulsivity. The study variables are related in the same way in men and women but boys show higher levels of preoccupied attachment, impulsivity, emotional dependence and BSD. This study gives us a clearer picture of how these variables are related and provides information that could be of great use in assisting people with BSD. This knowledge could be applied to improve both the treatment and prevention of this problem.

Buying-shopping disorder (BSD) is defined by an irresistible and intrusive preoccupation with purchasing items (Di Nicola et al., 2015). Individuals with this condition often spend excessive time shopping or planning purchases and report decreasing control over their behaviour, leading to adverse consequences for their psychosocial and financial functioning (Behar, 2018). Although this behaviour is not included in the *Diagnostic and Statistical Manual of Mental Disorders, Fifth Edition* ([DSM-5], American Psychiatric Association, 2013), most authors consider it a behavioural addiction (Müller, Laskowski et al., 2021). Other authors prefer to include it as an impulse control disorder (Behar, 2018). This does correspond to the categorisation of the *International Classification of Diseases* ([ICD-11] World Health Organization, 2018), where it is presented as an impulsive spectrum disorder.

A study by Müller, Laskowski et al. (2021) proposed diagnostic criteria for BSD through a Delphi study with 137 experts in the field from all over the world. The proposed diagnostic criteria are:

A) Persistent and/or recurrent dysfunctional buying/shopping-related behaviors, thoughts and related phenomena, as indicated by the following characteristics: (1) intrusive and/or irresistible urges and/or impulses and/or cravings and/or preoccupations for buying/shopping, (2) diminished control over buying/shopping, (3) excessive purchasing of items without utilizing them for their intended purposes, (4) use of buying/shopping to regulate internal states, (5) persistent and recurrent dysfunctional buying/shopping symptoms result in negative consequences and impairment in important areas of functioning and (6) reduction or cessation of excessive buying/shopping in negative emotional states and cognitive symptoms. B) Maintenance or escalation of dysfunctional buying/shopping behaviors despite negative consequences. C) The pattern of buying/shopping does not occur exclusively during a period of mania/hypomania. D) The pattern of buying/shopping is not better explained by the symptoms of another mental disorder (e.g., psychosis) or attributable to a medical condition (e.g., organic psychosyndrome) (p. 217).

Most authors were of the opinion that BSD is a behavioural addiction (Müller, Laskowski et al., 2021). This is consistent with the proposed diagnostic criteria. For example, it is noted that craving, lack of control over the behaviour and maintenance of the behaviour despite negative consequences is common in all addictions (APA, 2013). In general, the phenomenological parallels between buying-shopping disorder and behavioural addictions are supported (Trotzke et al., 2020).

BSD usually starts in adolescence or young adulthood (Medina et al., 2017) and is more common in women (Maraz et al., 2016). The incidence of BSD is on the rise in the context of the current pandemic (Covid-19). This situation has led to increased levels of stress, social isolation and leisure time, which may lead to increased BSD behaviours (Niedermoser et al., 2021). Increasing access to technology and the internet can trigger shopping addiction also in the digital world (Rose & Dhandayudham, 2014), due to the possibility of: (1) accessing a greater variety of products, with greater convenience (Niedermoser et al., 2021); (2) shopping anytime, anywhere, faster and without having to physically transport the purchased products (Kuss et al., 2018); (3) shopping without being observed, or interacting with other people. The dynamic nature of the medium (using pop-ups, personalised offers based on previous research, notifications, etc.) also generates frequent temptations and repeated stimulation, favouring cognitive overload and less self-control (Rose & Dhandayudham, 2014).

Different psychological factors could be involved in the aetiology of BSD such as: impulsivity, material values, depression, stress, low self-esteem, and difficulties in decision-making, among others (Zheng et al., 2020). One of those that has received the most attention has been impulsivity as it could be related to the loss of control experienced by people with BSD (De Paula et al., 2015; Rose & Segrist, 2014; De Sola-Gutiérrez et al., 2013) indicate that impulsivity is a very important element underlying addictive disorders and could act as a precursor to behavioural addictions in general.

Impulsivity is a multidimensional construct composed of three different aspects: cognitive impulsivity, which is related to restlessness of thought and rapid decision-making; motor impulsivity, which involves acting rashly without prior reflection; and unplanned impulsivity, which refers to performing actions without planning for the future (Salvo & Castro, 2013). Impulsivity has been found to be a predictor of BSD (Rose & Segrist, 2014).

Impulsivity is associated with emotional dependency (Castelló, 2005; Estévez et al., 2018). Emotional dependency can be defined as a pattern of unmet affective needs that attempt to be met maladaptively through interpersonal relationships (Urbiola et al., 2017). Emotionally dependent people share many characteristics of addictive pathologies and, in addition to the aforementioned loss of impulse control (Castelló, 2005; Estévez et al., 2018), present poor emotion regulation (Momeñe et al., 2017), comorbidity with anxious-depressive symptoms (Castelló, 2005), and they even often experience a “withdrawal syndrome” when they spend time apart from their partners (Castelló, 2012). The results of meta-analyses suggest that impulsivity may be a central process underlying all addictive disorders (Lee et al., 2019) and, consequently, comorbidity of different addictions is common (de Sola et al., 2013). Emotional dependence is related to several behavioural addictions, such as internet or mobile phone addiction (Estévez et al., 2017), and substance addiction (Momeñe et al., 2021). A study by Arda and Andriany (2019) indicates that consumers who have social relationships that do not meet their expectations and emotionally intimate relationships where they feel lonely tend to buy impulsively.

Emotional dependence could originate due to the relationship that these people maintain during their childhood with their primary caregivers, where there are affective deficiencies and unsatisfied socioemotional needs (Castelló, 2005, 2012). Therefore, certain attachment styles could be predictors of emotional dependence (Momeñe & Estévez, 2018). Three attachment styles can be distinguished in adolescence and adulthood: one secure attachment style and two insecure attachment styles (preoccupied and avoidant). Secure attachment style subjects are characterised by trust in themselves and others, share and express feelings openly, rate close experiences and interpersonal relationships as satisfying, and attach importance to family structure. Those with a preoccupied style are characterised by being continually worried about family relationships, have difficulty separating from loved ones, fear abandonment, show difficulties in personal autonomy and consider that they have difficulty overcoming experiences of loss. Finally, people with avoidant attachment reflect a defensive stance with regard to interpersonal relationships, emphasise personal strength to solve problems and define themselves as self-sufficient, independent and able to control their emotions (Main, 1990). Emotional dependence has been related, above all, to the preoccupied attachment style (Momeñe & Estévez, 2018). Similarly, dysfunctional family upbringing, with excessive parental authority and lack of affection in childhood, are possible predisposing factors for BSD (Behar, 2018).

Insecure attachment styles also correlate positively with impulsivity (Remondi et al., 2020) and this relationship could be mediated by emotional dependence (Estévez et al., 2018). However, there is little research on the role of impulsivity and the relationship between attachment styles and BSD (Müller, Claes et al., 2021). However, it has been shown that, in the case of technology addiction, impulsivity could mediate the relationship between this behavioural addiction and insecure attachment (Remondi et al., 2020).

Adolescence is a complex and highly vulnerable time of life, where impulsivity is one of the most characteristic elements, as the adolescent brain shows a maturational imbalance between the neurobiological systems underlying impulsivity (Kidd & Loxton, 2021). This imbalance results in a developmental period characterised by increased reward drive and weaker inhibitory control, which contributes to increased risk-taking behaviours during adolescence (Steinberg et al., 2018). This could increase behaviours such as BSD, which is particularly relevant in our social context. Also, during adolescence, social relationships acquire great importance and the first couple relationships are established (Chávez-Vera et al., 2018). Consequently, in adolescence, both emotional dependence (Martín & de la Villa Moral, 2019) and BSD may be present (Medina et al., 2017).

The levels of these variables are not the same for men and women. As mentioned, higher levels of BSD have been found in women (Estévez et al., 2021; Maraz et al., 2016). In contrast, men show higher levels of impulsivity (Navas et al., 2019) and emotional dependence (Martín & de la Villa Moral, 2019). Finally, higher levels of avoidant attachment (Medina et al., 2016) have been found in men and of preoccupied attachment in women, and the same in secure attachment (Padilla & Díaz, 2016).

This study pursues the following objectives: (1) To compare the types of attachment, levels of emotional dependence, impulsivity and BSD according to sex in the adolescent population; (2) To determine whether emotional dependence mediates the relationship between insecure attachment styles and BSD, while impulsivity moderates each of these relationships; (3) To analyse possible differences in this model in terms of the sample’s sex in the adolescent population.

Given the above, we expect to find the following: that emotional dependence mediates the relationship between insecure attachment styles (preoccupied and avoidant) and BSD, while impulsivity moderates each of these relationships (relationship between attachment and BSD, between attachment and emotional dependence, and between emotional dependence and BSD) (Fig. 1). No relationship between secure attachment and the rest of the variables is expected. Furthermore, it is hypothesised that the relationship of the variables will be the same for men and women. However, differences in the levels of some of the variables are expected according to the sex of the participants: women are expected to have higher levels of BSD and preoccupied attachment, while men are expected to have higher levels of impulsive avoidant attachment and emotional dependence. No differences are expected for secure attachment style.

## Method

### Participants

The sample was composed of 1498 Spanish-speaking adolescents of Ecuador whose age ranged from 14 to 18 years (*M* = 15.77, *SD* = 1.21). Concerning sex, 53.8% were men and 46.2% were women. Regarding their educational level, the four courses were balanced. Concretely, 24.4% of the participants were in tenth grade of primary education, 25.8% were in the first year of high school, 24.8% were in the second year of high school and 25% of the participants were in the third year of high school.

The design of the sample and the determination of its size were defined and calculated following the same criteria as those used in the Report of the Second National Survey of Secondary Education Students on Drug Use (2005) of the Republic of Ecuador conducted by the National Council for Narcotic and Psychotropic Substances Control. To calculate the ideal sample size, the confidence level (0.95) and the expected margin of error (0.015) between the results obtained in a sample and their generalisation to the population were considered. Finally, this sample size was increased to compensate for 10% of possible non-response. Each educational unit had the probability of selection directly proportional to the number of classes in the tenth grade of primary education and the first, second and third years of high school. The criteria for the selection of the strata were the representativeness criteria that correspond to the capital of Manabí of the Republic of Ecuador. This study represent 12 educational units and parishes.

### Instruments

#### Attachment styles

The attachment styles were measured with the Spanish brief version of the CaMir (Pierrehumbert et al., 1996), the CaMir-R (Balluerka et al., 2011). The CaMir-R is a questionnaire that measures attachment representations and conceptions of family functioning in adolescence and early adulthood. It consists of 32 items that the participant must answer on a 5-point Likert-type scale (1 = Totally disagree, 5 = Totally agree). The internal structure of the questionnaire consists of 7 dimensions, five of them referring to attachment representations (Security: Availability and Support of Attachment Figures; Family Concern; Parental Interference; Self-Reliance and Resentment against Parents; and Child Trauma) and the remaining two referring to representations of family structure (Value of Parental Authority and Parental Permissiveness). The attachment dimensions provide insight into the characteristics of attachment representations. In addition, four of these dimensions allow us to estimate the attachment style of the person: Secure, Preoccupied and Avoidant. Since this study has worked with attachment styles, only the results of four dimensions have been taken into account (Security: Availability and Support of Attachment Figures; Family Concern; Parental Interference; Self-Sufficiency and Resentment against Parents). Subjects with a T-score equal to or above 50 on the Security: Availability and Support of Attachment Figures dimension are considered to have a secure attachment style. Among subjects with an insecure attachment, the person will be classified as having a preoccupied or avoidant attachment style depending on their score on three dimensions. If the subject scores higher on the Family Concern and Parental Interference dimensions than on the Self-Reliance and Resentment against Parents dimension, he/she is considered to have an insecure preoccupied attachment style. If the opposite is true, with higher scores on the Self-sufficiency and resentment towards parents than on the Family Concern and Parental Interference dimensions, they are considered to have an avoidant attachment style.

The internal consistency of the Spanish adaptation ranged from 0.60 to 0.85 (Balluerka et al., 2011). In this study, the Cronbach’s alpha (α) of the subscales ranged from 0.68 to 0.90.

#### Emotional dependence

Emotional dependence was measured with the Emotional Dependence in Dating scale (DEN in Spanish; Urbiola et al., 2014). This scale is composed of 12 items that measure emotional dependence as the frequency with which the person feels: (1) the need to avoid being alone, referring to all those actions carried out for this purpose, (2) the need for exclusivity, referring to the need for their partner to be available only to them, (3) the need to please, actions carried out to please another person, ignoring their own needs, and (4) the development of an asymmetrical relationship, referring to the subordinate nature of the relationship. The questionnaire is rated on a 6-point Likert scale ranging from 0 (*never*) to 5 (*always*). Regarding the internal consistency, the alpha was 0.82 in the validation study (Urbiola et al., 2014). In this study, the alpha of the scale was 0.87.

#### Impulsivity

Impulsivity was assessed with the Barratt Impulsivity Scale (BIS 11) (Salvo & Castro, 2013). This 30-item scale measures impulsivity as a multidimensional construct with three subscales. It includes Cognitive Impulsivity, related to restlessness of thought and rapid decision-making; Motor Impulsivity, which involves acting rashly without prior reflection; and Unplanned Impulsivity, which refers to performing actions without planning for the future. Items are rated on a 4-point Likert scale ranging from 1 (*rarely or never*) to 4 (*always or almost always*). The internal consistency of the validation was 0.77 (Salvo & Castro, 2013). In this study, the reliability was α = 0.60.

#### Buying-shopping disorder

BSD was measured with the corresponding subscale of the MULTICAGE CAD 4 (Pedrero Pérez et al., 2007). It is a valid instrument for 14- to 90-year-olds. BSD is measured with four items on a dichotomous scale (yes/no). Categories are created based on the final sum of the scale which ranges from 0 to 4. Concretely, 0 or 1 is categorized as “Absence of BSD”, 2 as “Possible BSD” and 3 or 4 as “Quite likely or certain BSD”. The internal consistency (α = 0.79) and the test-retest reliability at 20 days of the Spanish validation were satisfactory (*r* = .89). The criterion validity was also adequate (it detected between 90 and 100% of the cases already diagnosed) (Pedrero Pérez et al., 2007). The reliability of the scale in the present study was α = 0.73.

### Procedure

Before starting the study, informed consent was requested from the parents and/or guardians of the adolescents who completed the questionnaires. They were also informed about the rules for completing the questionnaires, the duration and aspects to be measured, the voluntary nature of the study, the confidentiality and anonymity of the data obtained, and the contact details of the reference researcher. Given that data collection was carried out in paper format, during the administration of the questionnaires, the researcher remained in the classroom with the pupils until all the questionnaires had all been returned. The participants received a pencil and a certificate of participation as a token of appreciation. This study was conducted following the criteria of the Declaration of Helsinki (World Medical Association, 2013).

### Analytical procedure

Analyses were carried out with SPSS 27.0 (IBM Corp., 2020) and Mplus 8.0 (Muthén & Muthén, 2017). Preliminary analyses were conducted to analyze the patterns of missing data, the common method variance and the normality of the study variables. Only 0.6% of the data were missing, and the MCAR Little test was non-significant, χ^2^(517) = 390.67, *p* > .999, indicating that data were missing at random. Consequently, data were analyzed by pairwise deletion. Harman’s test was applied to test common method variance (Podsakoff et al., 2003). The results showed that the one factor solution explained only a 12.41% (< 50%) which supports the absence of relevant common method variance.

Two-sided Mardia multivariate skew and kurtosis tests were computed to test multivariate normality. This test determine the the global normality of all variables taking into account whether each variable in the data had a univariate normal distribution and whether each pair of variables had a bivariate normal distribution (Wulandari et al., 2021). Both the skew test (2.39, *p* < .001) and the kurtosis test (15.86, *p* = .010) were significant, indicating violation of the multivariate normality assumption. Therefore, Cook’s distance was applied to test potential influential multivariate outliers on both dependent variables (i.e., compulsive buying and emotional dependence). The results indicated that maximum Cook’s distance was 0.02 for compulsive buying and 0.03 for emotional dependence, as both values were lower than 1 (Cook & Weisberg, 1982), not influential multivariate outliers were identify. Therefore, the analyses were carried on with the full data set.

The analyses regarding hypotheses testing were developed in three steps. First, we tested the sex differences in the study variables. t-tests were conducted to analyze the sex differences in the continuous variables (i.e., emotional dependence and impulsivity), and chi-square in contingency tables to test the sex differences in the categorical variables (i.e., attachment styles and BSD). A value of Cohen’s *d* > 0.20 was considered small effect size; *d* > 0.50 medium effect size; and *d* > 0.80 large effect size (Cohen, 1988).

**Fig. 1:**
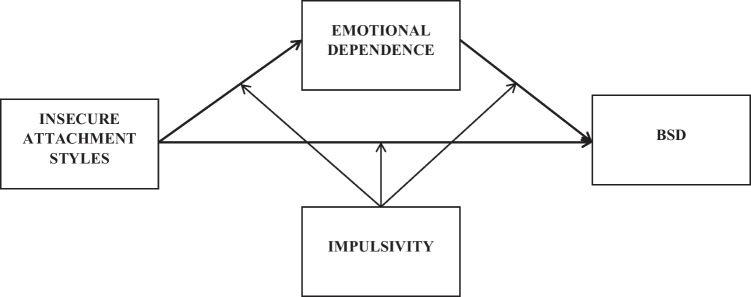
Hypothesized model

Second, the proposed baseline model was tested in the entire sample by path analysis in Mplus 8.0 (Muthén & Muthén, 2017) (Fig. 1). We applied weighted least squares mean and variance (WLSMV) estimation as it allows robust testing of relationships involving non-normal dependent categorical variables (Wang & Wang, 2012). Age and educational level were included as controls in the analyses. The adequacy of the model was calculated following Hu and Bentler’s (1999) criteria for model fit indicators. Non-significant chi-square, values of CFI and TLI equal to or greater than 0.90, and RMSEA and lower than 0.06 were considered indicators of good fit.

To examine the moderations, the direct effect of the independent variables on the dependent variables was included, and the variables of the products were mean-centered. Simple slopes were examined at the mean and ± 1.5 SD of the moderator variable. Model 1 and Model 7 of Stride et al.’s (2015) Mplus code were applied to test the moderation effects and the moderated indirect effects, respectively. The moderated indirect effect was calculated with 5,000 bootstrap random samples following Hayes’ and Scharkow’s (2013) suggestions for accurate computation of standard errors, confidence intervals, and the significance of the indirect effects.

Third, multi-group path analysis was performed to examine whether the structural paths were equivalent between men and women. To test structural path coefficient invariance, we compared the unconstrained model, where all path coefficients were freely estimated across the two groups, with a constrained model, where the corresponding structural paths were fixed to be equal across both groups. Comparisons of models were computed through the Wald test with the MODEL TEST command in Mplus because, due to the WLSMV estimation, model comparison of the two models cannot be directly computed by chi-square (Wang & Wang, 2012).

## Results

### Sex differences

Descriptive statistics, bivariate correlations, and sex differences of the continuous variables are displayed in Table 1. Results of the t-test indicated that men reported significantly higher emotional dependence and impulsivity than women, with medium and small effect sizes, respectively.

**Table 1:** Descriptive statistics, bivariate correlation and t-test results of sex differences in emotional dependence and impulsivity

	Full sample (*n* = 1498)		Women (*n* = 692)		Men (*n* = 806)				
Variable	*M*	*SD*	*M*	*SD*	*M*	*SD*	Correlation	*t*	*d*
Emotional dependence	1.18	0.96	0.93	0.85	1.40	1.01	0.14***	-9.69***	0.50
Impulsivity	2.21	0.28	2.18	0.29	2.24	0.28		-3.59***	0.21

*** p < .001

In Table 2, frequencies, percentages, and sex differences of the categorical variables are shown. Chi-square results indicated significant differences in attachment styles and BSD between men and women, whereas the effect sizes were non-significant and small, respectively. The comparison of the proportions of sexes indicated that women tend to be classified more often as secure-attached and less often as preoccupied-attached than men, whereas there were no significant differences in avoidant attachment. Regarding BSD, women reported the absence of BSD more frequently than men and probable and quite likely/certain BSD less frequently.

**Table 2:** Frequencies, percentages and chi-square results of sex differences in attachment styles and BSD

	Full sample (*n* = 1498)		Women (*n* = 692)		Men (*n* = 806)			
Variable	*n*	%	*n*	%	*n*	%	χ^2^	*d*
Attachment style							9.54**	0.16
Secure	895	59.7	442_a_	63.9	453_b_	56.2		
Preoccupied	190	12.7	75_a_	10.8	115_b_	14.3		
Avoidant	413	27.6	175_a_	25.3	238_a_	29.5		
BDS							42.15***	0.34
Absence	1045	69.8	540_a_	78.0	505_b_	62.7		
Probable	281	18.8	91_a_	13.2	190_b_	23.6		
Quite likely or certain	172	11.5	61_a_	8.8	111_b_	13.8		

Each subscript indicates a category in which the proportions of attachment/BSD do not differ significantly between sexes at the 0.05 level

** p < .01. *** p < .001

### Baseline model with entire sample

The path analysis was developed to test the hypothesized model in the entire sample. This initial model had a poor model fit, χ^2^(1) = 52.63, *p* < .001; CFI = 0.71, TLI = -3.77; RMSEA = 0.18, [0.14, 0.23]. Avoidant attachment had no significant effect on emotional dependence, nor did the interaction of this attachment style and impulsivity. Preoccupied attachment, its interaction with impulsivity, avoidant attachment, and the interaction of emotional dependence and impulsivity had no significant effects on BSD. These non-significant paths and avoidant attachment were deleted. The effect of avoidant attachment on BSD was maintained for the correct testing of the interaction effect, which was significant. This alternative model showed a good fit, χ^2^(4) = 2.26, *p* = .687; CFI = 1.00, TLI = 1.04; RMSEA = 0.00, [0.00, 0.03], so it was established as the baseline model.

**Fig. 2:**
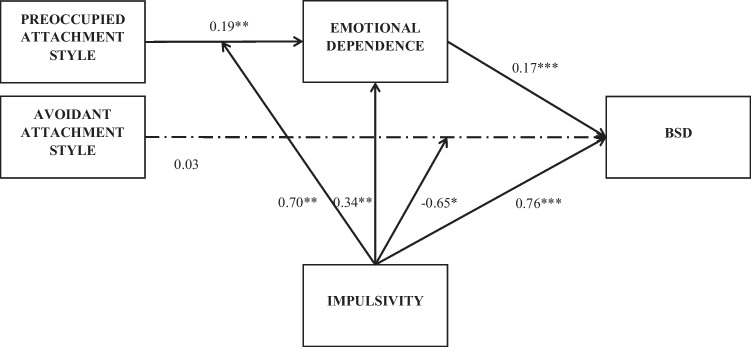
Path coefficients of the final model. The dashed line indicates a non-significant relationship. * *p* < .05. ** *p* < .01. *** *p* < .001

**Fig. 3:**
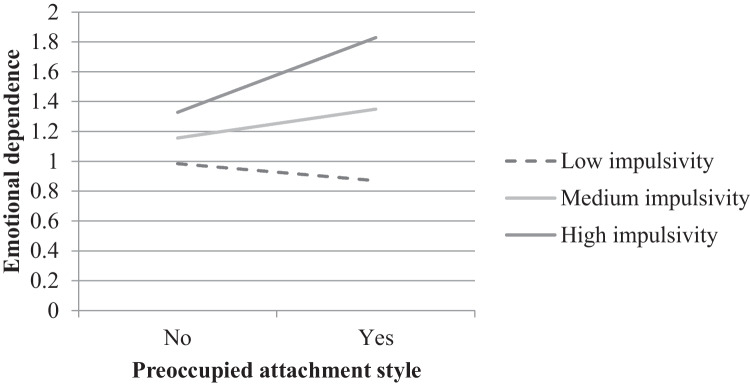
Moderation of the effect of preoccupied attachment style on emotional dependence by impulsivity

The path coefficients of the baseline model with the entire sample are displayed in Fig. 2. The interaction of preoccupied attachment and impulsivity on emotional dependence was significant. Concretely, preoccupied attachment style was only related to higher emotional dependence when impulsivity was medium (β = 0.19, *SE* = 0.07, *p* = .014) or high (β = 0.50, *SE* = 0.15, *p* = .001), but not when impulsivity was low (β = -0.11, *SE* = 0.15, *p* = .453) (see Fig. 3).

**Fig. 4:**
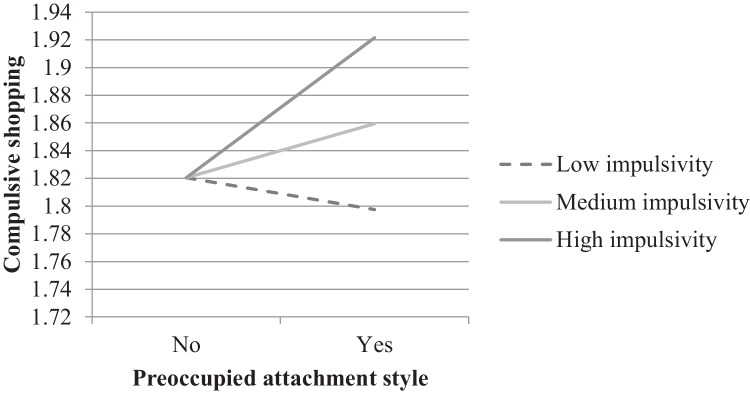
Indirect moderated effect of preoccupied attachment style on compulsive shopping through emotional dependence by impulsivity levels

Higher emotional dependence was significantly related to higher risk of BSD. The indirect effect of preoccupied attachment on BSD through emotional dependence was moderated by impulsivity. Specifically, preoccupied attachment style was indirectly related to higher risk of BSD due to emotional dependence only when the impulsivity levels were medium (Indirect Effect = 0.03, Bootstrap *SE* = 0.01, *p* = .456, 95% Bootstrap CI [< 0.01, 0.07]) or high (Indirect effect = 0.10, Bootstrap *SE* = 0.03, *p* = .002, 95% Bootstrap CI [0.03, 0.16]), but this indirect effect was not significant when impulsivity was low (Indirect effect = -0.02, Bootstrap *SE* = 0.03, *p* = .456, 95% Bootstrap CI [-0.08, 0.03]) (see Fig. 4).

**Fig. 5:**
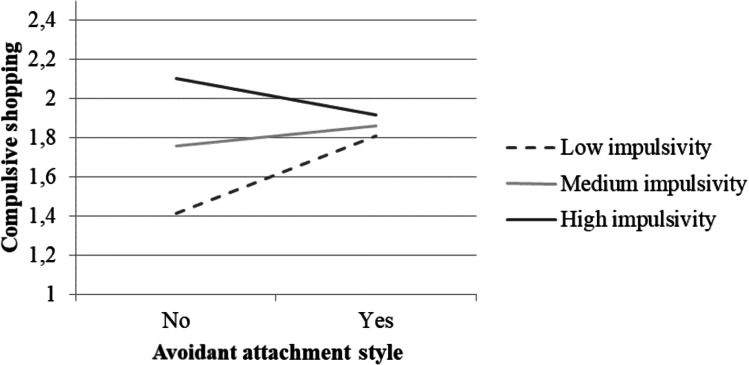
Moderation of the effect of avoidant attachment style on compulsive shopping by impulsivity

The interaction of avoidant attachment and impulsivity on BSD was significant. The simple slopes showed that an avoidant attachment style was related to higher risk of BSD only when impulsivity was low (β = 0.39, *SE* = 0.15, *p* = .012), but not when impulsivity was medium (β = 0.10, *SE* = 0.07, *p* = .183) or high (β = -0.18, *SE* = 0.13, *p* = .157) (see Fig. 5).

### Multi-group analysis between women and men

We developed multi-group analyses to compare the path coefficients between women and men. The unconstrained model, where paths coefficients were freely estimated for women and men fit the data well, χ^2^(8) = 3.01, *p* = .933; CFI = 1.00, TLI = 1.19; RMSEA = 0.00, [0.00, 0.01]. Similarly, the constrained model, where paths coefficients were fixed to be equal for both sexes, also showed adequate model fit, χ^2^(15) = 9.37, *p* = .856; CFI = 1.00 TLI = 1.11; RMSEA = 0.00, [0.00, 0.01]. The Wald test comparing the two models indicated that there were no significant differences between them,χ^2^(7) = 4.09, *p* = .769. This means that the free estimation of the parameters for women and men did not improve model fit, and the constrained model was a better model, as it is more parsimonious (i.e., fewer parameters are computed for the same model fit). These results indicated that there were no significant differences by sex in the paths of the baseline model and that the proposed model paths could be interpreted equally for women and men.

## Discussion

The first aim of the study was to analyse sex differences in the levels of attachment, emotional dependence, impulsivity, and BSD in adolescents. We found that men showed higher levels of preoccupied attachment, emotional dependence, impulsivity, and BSD. Women adolescents only showed higher levels of secure attachment. There were no significant differences in avoidant attachment. These findings are consistent with other studies that also found higher levels of emotional dependence (Martín & de la Villa Moral, 2019) in adolescent men. Other studies have found higher levels of impulsivity in young men than in young women (18–30 years old) (Navas et al., 2019), but no sex differences were found during adolescence (Li et al., 2019). Regarding differences in attachment styles in young people, Medina et al. (2016) found that men scored higher in avoidant attachment. Padilla and Díaz (2016) found a higher prevalence of preoccupied attachment in women and no sex differences in secure attachment. Finally, some authors report that BSD is higher in women (Estévez et al., 2021; Maraz et al., 2016). However, other studies in the area have found a similar prevalence among men and women, suggesting that the difference between the two sexes lies in the types of goods purchased. In this sense, it seems that women are more likely to purchase perfumery, cosmetics, jewellery, magazines or household goods, while men are more likely to purchase beverages, food, or electronic accessories (Maccarrone-Eaglen & Schofield, 2020). Nevertheless, discrepancies in the proportion of compulsive shoppers may stem from variation in dimensionality, in the screening tools employed, and/or in the demographic differences of the study samples (Maccarrone-Eaglen & Schofield, 2018).

The second objective of this study was to analyse whether the relational model proposed in the hypothesis was fulfilled: that emotional dependence mediates the relationship between attachment style and BSD, while impulsivity moderates each of these relationships (relationship between attachment and BSD, between attachment and emotional dependence, and between emotional dependence and BSD). The results show that the relational model found (Fig. 2) is similar to the initial model proposed. Analyses showed that a preoccupied attachment style was indirectly related to an increased risk of BSD due to emotional dependence only when impulsivity levels were medium or high because impulsivity moderates this indirect effect. No studies were found with which to compare these specific results. However, several studies have found a relationship between these variables. For example, it has been found that in young people, the preoccupied attachment style has the highest correlation with emotional dependence (Momeñe and Estévez, 2018), that impulsivity predicts BSD (de Paula et al., 2015) and is related to emotional dependence (Castelló, 2005), and that the relationship between attachment style and impulsive behaviour may be mediated by emotional dependence (Estévez et al., 2018).

The results also show that emotional dependence was positively related to the risk of BSD. Different studies have also found evidence for a positive relationship during adolescence between emotional dependence and addictions, both behavioural (Chávez-Vera et al., 2018) and substance addictions (Momeñe et al., 2021). A preoccupied attachment style was only related to greater emotional dependence when impulsivity was medium or high. It is concluded that with high and medium levels of impulsivity, the levels of purchase are higher than with low impulsivity. Among people with low impulsivity, those with avoidant attachment show a significantly higher risk of BSD. Kim and Koh (2018) showed that young people with avoidant attachment may show addictive behaviours in activities that are not related to interactions with other people (e.g., surfing the Internet). In general, insecure attachments have been shown to be risk factors for behavioural addictions (Monacis et al., 2017).

The third objective of the study was to analyse whether the proposed model was different for men and women. We concluded that there were no significant sex differences in the trajectories of the baseline model. Therefore, the trajectories of the proposed model could be interpreted equivalently for men and women. That is, although there are differences in the levels of the variables by sex, the way these variables are related is independent of the individual’s sex.

This study is not without limitations. As this is a cross-sectional study, it is not possible to establish causal relationships. We can only refer to relationships between variables. In turn, the results were obtained with self-administered questionnaires. Social desirability may have biased the data obtained because participants may have wanted to offer a positive self-image instead of answering sincerely. This is especially true given that this is an adolescent sample, which is an age where social desirability tends to be strongest. Moreover, attachment bonds are a complicated construct to measure. Some items refer to past experiences, so what is really being evaluated are the person’s constructions of such experiences.

It is concluded that with high and medium levels of impulsivity, the levels of BSD are higher than with low impulsivity and that emotional dependence is positively related to the risk of BSD. It should be noted that the results also show that a preoccupied attachment style is indirectly related to a higher risk of BSD due to emotional dependence when impulsivity levels are medium or high because this indirect effect is moderated by impulsivity. Among people with low impulsivity, those with avoidant attachment show a significantly higher risk of BSD. This study provides a clearer picture of how these variables are related. It offers information on the variables that influence BSD that can be very useful for working with people with BSD. This knowledge could be applied to improve both the treatment and prevention of this problem. On a practical level, for example, it has been shown that emotional dependence and impulsivity are risk factors for developing BSD. Considering that adolescence is characterised, among other things, by an increase in impusivity and the onset of relationships, these results justify the need to prevent both issues in adolescence. On the other hand, if a patient suffering from BSD comes to treatment, his or her attachment style, impusivity and emotional dependence should be worked on in order to help him or her manage the BSD.

The variables in the study are related in the same way in men and women. However, the results show that boys show higher levels of preoccupied attachment, impulsivity, emotional dependence, and BSD. This information is relevant as it is a reminder that adolescent men also suffer from these problems.

In future research, it might be interesting to analyse whether the relationship between these variables holds for young and adult participants, and it would also be interesting to analyse it in a clinical BSD population.

## Data Availability

The datasets generated during and/or analysed during the current study are available from the corresponding author on reasonable request.
